# Postpartum Depression in a High-Risk Population: An Exploratory Pilot Study Examining Family Composition Factors in Mothers With Substance Use During Pregnancy

**DOI:** 10.7759/cureus.94348

**Published:** 2025-10-11

**Authors:** Deepti Sanku, Caleb Zimmerman, Alan Ross, Beth Bailey

**Affiliations:** 1 College of Medicine, Central Michigan University, Mt. Pleasant, USA

**Keywords:** high-risk pregnancy, maternal mental health, maternal substance use, postpartum depression, vulnerable population

## Abstract

Background: Postpartum depression (PPD) affects approximately 10%-15% of mothers globally and represents a significant mental health concern. While most research shows first-time mothers are at higher risk for PPD, little is known about how family composition factors influence PPD risk in high-risk populations. This exploratory pilot study examined whether the presence of other children in the household and coparenting status predict PPD screening results among mothers with substance use during pregnancy.

Material and methods: This retrospective chart review included 101 mother-infant dyads from a university-affiliated pediatric practice in the Midwestern United States. All mothers had documented substance use during pregnancy, with the most commonly documented substances being marijuana (45.5%) and tobacco (36.6%). The majority (94.1%) were Medicaid recipients. PPD screening was conducted using the Edinburgh Postnatal Depression Scale (EPDS) during pediatric well-child visits within the first six months postpartum. Logistic regression analyses examined associations between PPD screening results and family composition factors (coparenting status and presence of other children).

Results: Among the 101 mothers screened, 28.7% screened positive for probable PPD. The prevalence was notably higher among mothers with other children (34.1%) compared to first-time mothers (15.8%). In adjusted models, mothers with other children had four times higher odds of screening positive for PPD (odds ratio(OR) = 4.09, 95% confidence interval (CI): 1.10-15.19), although the wide confidence interval indicates considerable uncertainty. Coparenting status was not significantly associated with PPD risk (adjusted OR = 1.02, 95% CI: 0.44-2.38).

Conclusions: This exploratory pilot study suggests that the relationship between family composition factors and PPD screening results may differ in high-risk populations compared to general samples. The association with multiparity warrants further investigation, although unmeasured confounding, particularly prior psychiatric history, limits causal interpretation. These preliminary findings highlight the need for larger prospective studies with comprehensive measurement of confounders to understand PPD risk factors in vulnerable populations and inform targeted interventions.

## Introduction

Postpartum depression (PPD) is a major depressive episode that occurs during pregnancy or within 12 months following delivery. The current Diagnostic and Statistical Manual of Mental Disorders, Fifth Edition (DSM-5) specifies PPD as major depressive disorder with peripartum onset, requiring the presence of five or more symptoms during a two-week period, with at least one symptom being either depressed mood or loss of interest or pleasure. These symptoms include the following: depressed mood most of the day, markedly diminished interest or pleasure in activities, significant weight loss or gain, insomnia or hypersomnia, psychomotor agitation or retardation, fatigue or loss of energy, feelings of worthlessness or excessive guilt, diminished ability to think or concentrate, and recurrent thoughts of death or suicidal ideation [1,2].

The global prevalence of PPD varies considerably, with recent systematic reviews reporting rates between 10% and 15% in high-income countries and higher rates in low- and middle-income countries [3]. Despite its high prevalence, PPD remains severely underdiagnosed, with studies suggesting that many cases go undetected in routine clinical practice. This underdiagnosis is particularly concerning given the substantial consequences of untreated PPD for both mothers and their children [4].

Consequences of untreated PPD

The impact of untreated PPD extends beyond the immediate postpartum period and affects multiple domains of maternal and child well-being. For mothers, untreated PPD is associated with chronic depressive episodes, physical health consequences, including chronic pain syndromes, and reduced physical functioning [5]. The psychological impacts encompass anxiety disorders, reduced self-efficacy in parenting, and impaired mother-infant bonding [5,6]. In severe cases, there is an increased risk of suicide, which accounts for approximately 20% of postpartum maternal deaths [7].

For infants and children, maternal PPD disrupts critical developmental processes through multiple pathways. The impairment in mother-infant interaction patterns characteristic of PPD interferes with the development of secure attachment relationships. Research has demonstrated that infants of mothers with PPD show increased negative emotionality, difficult temperament, and dysregulated stress responses. Long-term follow-up studies reveal associations between maternal PPD and children's cognitive delays, language impairments, and behavioral problems [5,8].

Current understanding of risk factors

The etiology of PPD is multifactorial, involving complex interactions between biological, psychological, and social factors [8-10]. Previous research has identified several categories of risk factors, including prenatal depression and anxiety, prior history of depression, recent stressful life events, and poor social support [10-14]. Notably, a history of depression or psychiatric illness, whether prior to pregnancy or during previous postpartum periods, represents one of the most significant predictors of PPD [10,13]. It is important to acknowledge that depression, anxiety, other mental disorders, stressful life events, poor social support, and low socioeconomic status are also factors that contribute to substance use during pregnancy [10,15]. These overlapping risk factors create complex relationships between substance use and PPD that warrant consideration when interpreting findings in high-risk populations. However, the specific role of family composition factors remains less well understood, particularly in populations with substance use during pregnancy.

The role of family composition

While social support has been extensively studied as a protective factor against PPD [12], the specific role of family composition factors remains less well understood. The impact of having multiple children on PPD risk shows mixed findings in the literature. Most research has found that primiparous women (first-time mothers) are at higher risk for PPD compared to multiparous women [16,17], while some studies find that first-time mothers and multiparous mothers are at equal risk [18]. Interestingly, one study found that low social support significantly increased PPD risk for multiparous women, but not for first-time mothers, suggesting that the relationship between risk factors and PPD may differ by parity [19]. However, research specifically examining parity and PPD in high-risk populations, such as mothers with substance use during pregnancy, remains limited.

Theoretical frameworks suggest that caring for multiple children could increase PPD risk through several mechanisms: increased physical demands and fatigue, reduced time for self-care and recovery, competing demands for attention that may intensify feelings of inadequacy, and financial strain associated with larger family size. These mixed empirical findings and competing theoretical perspectives highlight the need for further research, particularly in high-risk populations where the balance of protective and risk factors may differ from general postpartum populations. In vulnerable populations facing resource constraints, the typical protective effects of parenting experience may be outweighed by the burden of caring for multiple children without adequate support [19-21].

Special considerations for a high-risk population

Mothers who use substances during pregnancy are especially vulnerable to postpartum depression (PPD), with studies indicating that approximately 29% of pregnant women who use substances experience PPD, a rate significantly higher than that of the general postpartum population [22]. This increased risk reflects the complex interplay of mental health vulnerabilities, stressful life events, poor social support, and low socioeconomic status, all of which are established contributors to both substance use and PPD [10,15]. Notably, recent research has shown that maternal depression is one of the strongest predictors of substance use during pregnancy, increasing the likelihood of alcohol, tobacco, and cannabis use [10]. The relationship between these risk factors can be particularly complex in high-risk groups. While previous parenting experience may reduce the risk of PPD in some populations, large studies have shown that this protective effect is less consistent when mothers face additional stressors such as poverty or limited support systems [17]. In fact, multiparous women with low social support in resource-constrained environments face especially high odds of developing PPD compared to those with stronger support networks [19]. These findings highlight the need to consider how risk factors interact in high-risk populations and support the development of targeted interventions [17,19].

Study aims

Given the limited evidence regarding family composition factors and PPD in high-risk populations, this exploratory pilot study aimed to examine whether the presence of other children in the household and coparenting status predict PPD risk among mothers with documented substance use during pregnancy. We hypothesized that both parenting alone and having multiple children would be associated with increased PPD risk in this vulnerable population.

## Materials and methods

Study design and setting

This exploratory pilot study utilized a retrospective chart review of electronic medical record data from mother-infant dyads receiving care at a university-affiliated pediatric practice in the Upper Midwest region of the United States. The practice primarily serves a rural and socioeconomically disadvantaged population. The study was reviewed and approved by the appropriate Institutional Review Board, which determined it to be of minimal risk. A waiver of informed consent was granted because of the retrospective design and minimal risk to participants. The single-site, retrospective design and focus on a specific high-risk population (mothers with substance use during pregnancy) limit generalizability, as discussed in the Limitations section.

Participants

Inclusion Criteria

Participants were mothers whose infants (born between January 2018 and December 2020) received pediatric care at the study site. To be included, mothers must have (1) delivered at the affiliated university hospital, (2) received prenatal care at an affiliated obstetrics practice, (3) had documented information about substance use during pregnancy in their medical record, and (4) completed at least one Edinburgh Postnatal Depression Scale (EPDS) screening within six months postpartum during a pediatric well-child visit.

Exclusion Criteria

Mothers were excluded if they (1) had incomplete medical records regarding pregnancy substance use, (2) had no documented EPDS screening results, (3) had missing coparenting status information, (4) experienced fetal or infant death, or (5) had infants with major congenital anomalies requiring intensive medical intervention.

Sample characteristics

The initial sampling frame consisted of 140 mother-infant dyads. After applying inclusion and exclusion criteria, 39 (27.9%) of the 140 participants were removed, resulting in a final analytical sample of 101 mothers.

All mothers in the sample had documented substance use during pregnancy, which was an inclusion criterion for this pilot study. However, for the purposes of analysis, we specifically examined marijuana and tobacco use, which were the most commonly documented substances in our sample. The sample was characterized by high rates of marijuana use (45.5%) and tobacco use (36.6%), with mothers able to use one or both substances (categories are not mutually exclusive). The sample also reflected low socioeconomic status (94.1% receiving Medicaid) and relatively young maternal age (mean = 25.2 years, standard deviation (SD) = 5.4).

Documentation of substance use varied in detail across medical records. Some records included information on frequency, dose, and timing during pregnancy, while others contained only binary (yes/no) indicators. This variability limited our ability to differentiate between occasional use, abuse, or dependence, or to examine dose-response relationships.

Primary outcome: Postpartum depression

PPD was assessed using the Edinburgh Postnatal Depression Scale (EPDS), a 10-item self-report questionnaire specifically validated for detecting depression in the perinatal period [23]. The EPDS assesses depressive symptoms experienced in the past seven days, with items scored 0-3 and total scores ranging from 0 to 30. For this pilot study, we used a cutoff score of ≥10 to indicate probable PPD requiring further clinical evaluation. This threshold was selected based on validation studies showing adequate sensitivity and specificity for detecting depression in postpartum populations [23-25]. While a cutoff of ≥13 is sometimes used for probable major depression, we chose the lower threshold to maximize sensitivity for identifying at-risk mothers in this vulnerable population, consistent with recommendations for clinical screening programs [25]. It is important to note that the EPDS is a screening instrument, not a diagnostic tool, and scores above the threshold indicate the need for further evaluation by a healthcare professional rather than constituting a clinical diagnosis of major depressive disorder.

Primary predictors: Family composition variables

Two family composition variables and substance use patterns were examined in this exploratory study.

Coparenting status was determined from demographic information in the medical record, with mothers classified as "Coparenting" if they were married to or cohabitating with the infant's biological father at the time of delivery. Mothers were classified as "Parenting Alone" if they were single, separated, divorced, or not living with the infant's father. This binary classification does not capture the quality of the coparenting relationship or the quality of social support available to mothers, which represents a limitation of this study.

The presence of other children was extracted from parity information and household composition data. Mothers were classified as "First-Time Mothers" if the index pregnancy was their first live birth with no other children in the household, while mothers were classified as "Multiple Children" if they had one or more other children living in the household.

Covariates

The following variables were extracted to characterize the sample and control for potential confounding: maternal age at delivery (recorded as a continuous variable in years) and insurance status at delivery (categorized as Medicaid/uninsured versus private insurance, serving as a proxy for socioeconomic status).

Unmeasured confounding variables

Due to the retrospective design and reliance on available medical record data, several important established risk factors for PPD were not measured in this pilot study. These unmeasured variables include the following: prior history of depression or anxiety disorders, previous PPD during prior pregnancies, history of intimate partner violence or other stressful life events, quality of social support beyond binary coparenting status, and presence of other comorbid mental disorders. While some of this information was present in certain medical records, the documentation was inconsistent and not standardized across all records, precluding systematic extraction and reliable analysis. The absence of data on these key confounders represents a significant limitation and raises concerns about residual confounding, as these factors could account for or modify the observed associations between family composition variables and PPD risk. This limitation is particularly important given that depression, anxiety, and other mental disorders commonly co-occur with substance use during pregnancy, and prior depression is one of the strongest predictors of PPD [10,13,15]. The potential impact of these unmeasured confounders on our findings is discussed in detail in the Limitations section.

Statistical analysis

All statistical analyses followed established guidelines with two-tailed tests employed and significance defined at p < 0.05 unless indicated otherwise. A priori estimates suggested that a modest number of participants per group would be required to achieve adequate power for this exploratory pilot study. Our final analytic sample size of 101 mothers was adequate for examining the primary research questions regarding family composition factors and PPD risk.

Descriptive and Bivariate Analyses

Descriptive statistics characterized the sample using means and standard deviations for continuous variables and frequencies with percentages for categorical variables. Bivariate associations between family composition variables and covariates were examined using independent samples t-tests for continuous variables and chi-square tests for categorical variables. Variables associated with family composition at p < 0.10 were retained as potential confounders for multivariable analyses.

Multivariable Analyses

Logistic regression models examined associations between family composition variables and PPD. We constructed three models for each predictor: the unadjusted model examined raw associations, Model 1 adjusted for significant covariates (maternal age and insurance status), and Model 2 adjusted for covariates and included both family composition variables. Results are presented as odds ratios (OR) with 95% confidence intervals (CI).

## Results

Sample characteristics

Among the 101 mothers included in the analysis, the mean age at delivery was 25.2 years (SD = 5.4, range: 18-39). The majority were Medicaid recipients (n = 95/101, 94.1%), reflecting the low socioeconomic status of the sample. Regarding family composition, 25/101 (24.8%) were coparenting with the infant's father, while 76/101 (75.2%) were parenting alone. Moreover, 19/101 (18.8%) were first-time mothers, while 82/101 (81.2%) had other children in the household.

All mothers in the sample had documented substance use during pregnancy per the inclusion criteria. Specifically examining marijuana and tobacco use, 37/101 (36.6%) used tobacco during pregnancy (either alone or in combination with other substances), and 46/101 (45.5%) used marijuana during pregnancy (either alone or in combination with other substances). These categories are not mutually exclusive, as some mothers used both marijuana and tobacco.

Prevalence of postpartum depression

Using the EPDS cutoff of ≥10, 29/101 (28.7%) mothers screened positive for probable PPD. This prevalence is approximately double the rate typically reported in general postpartum populations. The mean EPDS score was 7.7 (SD = 5.2), with scores ranging from 0 to 24.

Bivariate associations with family composition

Mothers parenting alone were significantly younger at delivery (mean: 24.4 versus 26.6 years, t = 2.19, p = 0.03) compared to coparenting mothers. The distribution of background factors by coparenting status is presented in Table 1.

**Table 1:** Background factors by parenting status Values are represented as number (%) for categorical variables or mean (SD) for continuous variables. t = t-test statistic, χ² = chi-square test statistic, p = p-value for bivariate comparisons between parenting groups SD: standard deviation

Background factor	Parenting alone (n = 76)	Parenting with partner (n = 25)	t/χ²	p
White, non-Hispanic race	35 (46.0%)	15 (60.0%)	1.78	0.182
Maternal age at delivery, mean (SD)	24.4 (5.3)	26.6 (5.5)	2.19	0.03
Medicaid/uninsured	73 (96.0%)	22 (88.0%)	3.38	0.066
First-time mothers	13 (17.1%)	6 (24.0%)	1.13	0.288
Tobacco use during pregnancy	25 (32.9%)	12 (48.0%)	2.23	0.136
Marijuana use during pregnancy	36 (47.4%)	10 (40.0%)	0.55	0.457

Association between family composition and PPD

The prevalence of probable PPD did not differ significantly between mothers coparenting (7/25 (28.0%)) and those parenting alone (24/76 (31.6%)). However, a notable difference emerged based on the presence of other children: 28/82 (34.1%) of mothers with multiple children screened positive for PPD compared to only 3/19 (15.8%) of first-time mothers. The results from logistic regression analyses are presented in Table 2.

**Table 2:** Prediction of PPD based on parenting and number of children Values represent OR with 95% confidence intervals in parentheses. "Ref" indicates the reference category for each predictor. Dashes (-) indicate category headers with no direct numerical value. PPD prevalence is shown as number positive/total (%). Model 1 adjusts for maternal age at delivery and insurance status (Medicaid/uninsured versus private). Model 2 adjusts for covariates and includes both family composition variables. PPD: postpartum depression, OR: odds ratios

Predictor	PPD prevalence	Unadjusted OR	Model 1	Model 2
Parenting status	-	-	-	-
Coparenting	7/25 (28.0%)	Ref	Ref	Ref
Parenting alone	24/76 (31.6%)	1.24 (0.56-2.75)	1.15 (0.51-2.63)	1.02 (0.44-2.38)
Number of children	-	-	-	-
First-time mother	3/19 (15.8%)	Ref	Ref	Ref
Multiple children	28/82 (34.1%)	3.72 (1.04-13.35)	4.07 (1.10-15.02)	4.09 (1.10-15.19)

In unadjusted analyses, mothers with multiple children had 3.72 times higher odds of PPD compared to first-time mothers (95% CI: 1.04-13.35). This association strengthened slightly after adjusting for maternal age and insurance status (Model 1: OR = 4.07, 95% CI: 1.10-15.02) and remained significant in the fully adjusted model including both family composition variables (Model 2: OR = 4.09, 95% CI: 1.10-15.19).

## Discussion

This exploratory pilot study examined the relationship between family composition factors and PPD screening results in a high-risk population characterized by substance use during pregnancy and socioeconomic disadvantage. Our findings suggest that having multiple children was associated with increased odds of screening positive for PPD, while coparenting status showed no significant association. These preliminary findings provide insight into how PPD risk factors may operate differently in vulnerable populations compared to general postpartum samples, while guiding future research directions. Important limitations regarding unmeasured confounding must be considered when interpreting these results.

Findings in the context of existing literature

Our finding that mothers with multiple children had higher rates of positive PPD screening than first-time mothers differs from patterns typically observed in general postpartum populations. Most studies of general postpartum samples have shown that first-time mothers are at higher risk for PPD, with some research demonstrating that multiparous women show lower risk than first-time mothers [16,17]. This difference raises important questions about whether risk factor patterns vary across populations with different levels of resources and support.

Several explanations merit consideration. First, we did not assess prior history of depression or anxiety disorders, nor whether multiparous mothers had experienced PPD during previous pregnancies. Given that prior depression is one of the strongest predictors of PPD [10,13], and prior PPD substantially increases risk of recurrence [26], mothers with chronic or recurrent depression may be more likely to have multiple children and to screen positive for current PPD. This would create an apparent association between multiparity and PPD that actually reflects recurrent depressive episodes.

Alternatively, our sample's unique characteristics (94.1% Medicaid recipients, universal substance use, and rural residence with limited resources) may create conditions where the typical protective effects of parenting experience are outweighed by resource constraints and cumulative stressors. In populations with adequate support, previous parenting experience and established support networks may be protective [17]. However, in resource-constrained environments where mothers lack access to childcare assistance, household help, and other services, the burden of multiple children may overwhelm any benefits of prior experience [19-21]. Without measuring key confounders, we cannot definitively distinguish between these explanations in this pilot study.

Potential mechanisms in resource-constrained populations

If the association we observed reflects a true phenomenon specific to high-risk populations rather than confounding, it would align with theories of maternal role strain and resource depletion. Caring for multiple children while recovering from childbirth creates competing demands that may overwhelm maternal coping resources, particularly where socioeconomic disadvantage limits access to support services. Physical demands may exacerbate postpartum fatigue, sleep deprivation may be more severe when attending to multiple children with different schedules, and the emotional demands of dividing attention may intensify feelings of inadequacy [20,21].

Understanding these potential mechanisms is important for developing interventions tailored to vulnerable populations. Mothers facing substance use challenges, poverty, and limited social support may experience the postpartum period very differently from mothers in general population studies and may require different types and intensities of support.

Coparenting status and the complexity of social support

Coparenting status was not associated with PPD risk in our pilot sample. While some research shows single mothers experience higher rates of depression [27], other studies have found that single mothers do not necessarily demonstrate elevated risk for postpartum depression specifically [28]. This finding likely reflects the limitations of our binary measure (married/cohabitating versus not) rather than suggesting social support is irrelevant. What matters most is probably relationship quality, actual support provided, and presence of conflict or violence, none of which our measure captured. A partner who is present but unsupportive or abusive may offer no protective benefit, while some single mothers may have strong support from family or friends. This highlights the need for more nuanced measurement of social support in future research with vulnerable populations.

Statistical precision and uncertainty

The wide confidence interval (1.10-15.19) for the association between multiple children and PPD screening indicates considerable uncertainty about the magnitude of any effect. The lower bound suggests any true association could be quite modest, while the upper bound suggests it could be substantial. This imprecision reflects our modest pilot sample size (N = 101) and underscores the preliminary nature of our findings. Larger studies are needed to obtain more precise estimates.

Implications for research and practice

This exploratory pilot study highlights the importance of conducting PPD research specifically within vulnerable populations. Risk factor patterns identified in general postpartum samples may not fully apply to mothers facing multiple concurrent stressors such as substance use, poverty, and limited access to resources. Understanding how PPD risk factors operate in these populations is essential for developing targeted interventions and support services for those who need them most.

From a clinical standpoint, the preliminary findings from this pilot study do not support immediate changes to screening protocols. Universal PPD screening for all postpartum women remains the evidence-based recommendation, regardless of parity, substance use history, or other risk factors [29]. However, if future prospective studies with comprehensive measurement of confounders confirm that caring for multiple children poses unique challenges in resource-constrained environments, this could inform the development of tailored support programs. Interventions providing respite care, household assistance, or parent education focused on managing multiple children's needs with limited resources could theoretically address specific challenges faced by vulnerable mothers, although such intervention development should await more definitive evidence from larger-scale studies.

Strengths and limitations

This exploratory pilot study has several strengths, including the use of a validated screening instrument administered during routine pediatric care, focus on an understudied and vulnerable population, and transparent reporting of methods and limitations. However, as a pilot study, substantial limitations must be acknowledged.

Unmeasured Confounding

We did not measure several established risk factors for PPD, including prior history of depression or anxiety, previous PPD during prior pregnancies, quality of social support beyond binary coparenting status, history of intimate partner violence, or other comorbid mental disorders [10,12,13]. While some of this information was present in certain medical records, the documentation was inconsistent and not standardized across all records, precluding systematic extraction and reliable analysis. The observed association between multiple children and PPD screening could be partially or entirely explained by these unmeasured variables, particularly prior psychiatric history.

Statistical Precision

While the association between multiple children and PPD screening was statistically significant, the wide confidence interval (1.10-15.19) indicates considerable uncertainty about the magnitude of the effect. The lower bound suggests the true association could be modest, while the upper bound suggests it could be substantial. This imprecision reflects our modest pilot sample size (N = 101) and underscores the need for larger studies to obtain more precise estimates and confirm the magnitude of this association.

Substance Use Measurement

Documentation of substance use varied in detail across medical records. Some records included information on frequency, dose, timing during pregnancy, and duration, while others contained only binary (yes/no) indicators. This variability in documentation detail limited our ability to examine dose-response relationships, differentiate between occasional use versus abuse or dependence, or stratify by substance type or severity.

Design and Sampling

The retrospective, single-site design limits causal inference and generalizability. We excluded 27.9% of potential participants (39 of 140) due to not meeting the inclusion criteria. If excluded mothers differed systematically from included mothers regarding both family composition and PPD risk, selection bias may affect our findings.

EPDS Limitations

The EPDS is a screening instrument that indicates the need for further evaluation, not a diagnostic tool providing confirmed diagnoses [23]. Our prevalence estimate of 28.7% represents mothers at elevated risk rather than confirmed PPD cases. Additionally, EPDS administration at varying times within the first six months postpartum introduces temporal variability, as PPD risk and symptom severity change over the postpartum period [4].

Pilot Study Limitations

As an exploratory pilot study, this work was designed to generate hypotheses and assess feasibility rather than provide definitive answers. The modest sample size, single-site design, and lack of comprehensive confounder measurement limit our ability to draw firm conclusions about causality or generalizability.

Generalizability

Findings are specific to mothers with substance use during pregnancy, very low socioeconomic status (94.1% receiving Medicaid), and rural residence in the Midwestern United States. The relationship between parity and PPD may differ substantially in populations without substance use, in higher-income populations with greater access to childcare and support services, or in urban settings with different resources.

Future directions

Several research priorities emerge from this exploratory pilot work. Prospective, multi-site studies including diverse vulnerable populations are needed to determine whether our preliminary findings replicate in other high-risk samples. Such studies must include a comprehensive baseline assessment of psychiatric history, particularly prior depression and previous PPD episodes, to address the confounding that limits interpretation of our pilot findings. Studies should measure depression at standardized postpartum time points using both screening instruments and structured diagnostic interviews.

Future research should also include validated substance use disorder assessments that distinguish occasional use from abuse and dependence, comprehensive measures of relationship quality and social support (not just partner presence), and screening for intimate partner violence. This would allow researchers to disentangle complex interrelationships among risk factors in vulnerable populations.

If future larger-scale studies confirm that caring for multiple children in resource-constrained environments contributes to PPD risk in mothers with substance use histories, independent of prior psychiatric history, investigation of underlying mechanisms would be valuable. Studies could examine whether associations differ by the availability of childcare support, household assistance, financial resources, and other supports. This understanding could inform the development of targeted interventions for vulnerable mothers with multiple children facing substance use challenges.

## Conclusions

This exploratory pilot study examined family composition factors and PPD screening results in 101 mothers with substance use during pregnancy and low socioeconomic status. We found that having multiple children was associated with increased odds of positive PPD screening compared to first-time mothers, a pattern that differs from general postpartum populations, where first-time mothers typically show elevated risk. However, these findings must be interpreted with caution, given the study's exploratory nature, modest sample size, and significant unmeasured confounding, particularly the absence of data on prior psychiatric history. As a pilot study, this work was designed to generate hypotheses for future investigation rather than provide definitive conclusions. Our findings suggest that examining family composition factors in vulnerable populations is important and warrants further investigation, as risk patterns may differ substantially from those observed in general postpartum samples. Universal PPD screening remains essential for all postpartum women regardless of parity or other characteristics.

Building on these preliminary observations, larger prospective, multi-site studies are needed to confirm whether parity influences PPD risk differently in vulnerable populations. Such studies should include comprehensive baseline measurement of prior psychiatric history, validated substance use disorder assessments, and measures of relationship quality and social support. Only with more rigorous research addressing the limitations identified in this pilot work can we develop evidence-based screening and intervention strategies tailored to high-risk mothers. Given the substantial burden of PPD and the complex needs of mothers facing substance use and socioeconomic challenges, advancing this research agenda is critical for improving maternal mental health outcomes in vulnerable populations.

## References

[1] American Psychiatric Association, DSM-5 Task Force (2013). Diagnostic and Statistical Manual of Mental Disorders: DSM-5™ (5th Ed.).

[2] Bains N, Abdijadid S (2023). Major depressive disorder. StatPearls [Internet].

[3] Wang Z, Liu J, Shuai H (2021). Mapping global prevalence of depression among postpartum women. Transl Psychiatry.

[4] Carlson K, Mughal S, Azhar Y (2025). Perinatal depression. StatPearls [Internet].

[5] Slomian J, Honvo G, Emonts P, Reginster JY, Bruyère O (2019). Consequences of maternal postpartum depression: a systematic review of maternal and infant outcomes. Womens Health (Lond).

[6] Sit DK, Wisner KL (2009). Identification of postpartum depression. Clin Obstet Gynecol.

[7] Lindahl V, Pearson JL, Colpe L (2005). Prevalence of suicidality during pregnancy and the postpartum. Arch Womens Ment Health.

[8] Payne JL, Maguire J (2019). Pathophysiological mechanisms implicated in postpartum depression. Front Neuroendocrinol.

[9] Seltzer A (1980). Postpartum mental syndromes. Can Fam Physician.

[10] Yim IS, Tanner Stapleton LR, Guardino CM, Hahn-Holbrook J, Dunkel Schetter C (2015). Biological and psychosocial predictors of postpartum depression: systematic review and call for integration. Annu Rev Clin Psychol.

[11] Solikhah FK, Nursalam N, Subekti I, Winarni S, Yudiernawati A (2022). Determination of factors affecting post-partum depression in primary healthcare during the COVID-19 pandemic. J Public Health Afr.

[12] Zhao XH, Zhang ZH (2020). Risk factors for postpartum depression: an evidence-based systematic review of systematic reviews and meta-analyses. Asian J Psychiatr.

[13] Agrawal I, Mehendale AM, Malhotra R (2022). Risk factors of postpartum depression. Cureus.

[14] Stewart DE, Vigod SN (2019). Postpartum depression: pathophysiology, treatment, and emerging therapeutics. Annu Rev Med.

[15] Brown RA, Dakkak H, Gilliland J, Seabrook JA (2019). Predictors of drug use during pregnancy: the relative effects of socioeconomic, demographic, and mental health risk factors. J Neonatal Perinatal Med.

[16] Raoufi F, Foroughi Kaldareh Z (2025). A comparative study of postpartum depression and psychological hardiness between first-time and multiparous mothers. Int J Body Mind Cult.

[17] Bradshaw H, Riddle JN, Salimgaraev R, Zhaunova L, Payne JL (2022). Risk factors associated with postpartum depressive symptoms: a multinational study. J Affect Disord.

[18] Zhang J, Wang P, Fan W, Lin C (2024). Comparing the prevalence and influencing factors of postpartum depression in primiparous and multiparous women in China. Front Psychiatry.

[19] Cho H, Lee K, Choi E (2022). Association between social support and postpartum depression. Sci Rep.

[20] Saharoy R, Potdukhe A, Wanjari M, Taksande AB (2023). Postpartum depression and maternal care: exploring the complex effects on mothers and infants. Cureus.

[21] Parkes A, Sweeting H, Wight D (2015). Parenting stress and parent support among mothers with high and low education. J Fam Psychol.

[22] Pacho M, Aymerich C, Pedruzo B (2023). Substance use during pregnancy and risk of postpartum depression: a systematic review and meta-analysis. Front Psychiatry.

[23] Cox JL, Holden JM, Sagovsky R (1987). Detection of postnatal depression. Development of the 10-item Edinburgh Postnatal Depression Scale. Br J Psychiatry.

[24] Chorwe-Sungani G, Chipps J (2017). A systematic review of screening instruments for depression for use in antenatal services in low resource settings. BMC Psychiatry.

[25] Haßdenteufel K, Lingenfelder K, Schwarze CE (2021). Evaluation of repeated web-based screening for predicting postpartum depression: prospective cohort study. JMIR Ment Health.

[26] Rasmussen MH, Strøm M, Wohlfahrt J, Videbech P, Melbye M (2017). Risk, treatment duration, and recurrence risk of postpartum affective disorder in women with no prior psychiatric history: a population-based cohort study. PLoS Med.

[27] Kareem OM, Oduoye MO, Bhattacharjee P (2024). Single parenthood and depression: a thorough review of current understanding. Health Sci Rep.

[28] Agnafors S, Bladh M, Svedin CG, Sydsjö G (2019). Mental health in young mothers, single mothers and their children. BMC Psychiatry.

[29] (2025). Postpartum Support International: Screening recommendations. https://postpartum.net/professionals/screening/.

